# Clinical Outcomes and Quality of Life of Patients Receiving Multi-Solid-Organ Transplants in Childhood Are Excellent: Results From a 20-Year Cohort Study

**DOI:** 10.3389/ti.2024.13372

**Published:** 2024-08-14

**Authors:** Alicia Paessler, Hannah Maple, Miriam Cortes, Jacob Simmonds, Yincent Tse, Maduri Raja, Mordi Muorah, Nicos Kessaris, Jelena Stojanovic

**Affiliations:** ^1^ Great Ormond Street Hospital for Children NHS Foundation Trust, London, United Kingdom; ^2^ Guy’s and St. Thomas’ NHS Foundation Trust, London, United Kingdom; ^3^ King’s College Hospital NHS Foundation Trust, London, United Kingdom; ^4^ Newcastle Upon Tyne Hospitals NHS Foundation Trust, Newcastle upon Tyne, United Kingdom; ^5^ Birmingham Women’s and Children’s NHS Foundation Trust, Birmingham, United Kingdom; ^6^ UCL Institute of Child Health, London, United Kingdom

**Keywords:** paediatric, multi-organ transplant, kidney transplant, liver transplant, heart transplant

## Abstract

Advances in medicine allow children with previously fatal conditions to survive longer and present as transplant candidates; some requiring multiple solid-organ transplants (MSOT). There is limited data on clinical outcomes and no data on quality of life (QoL). In this mixed methods cohort study clinical outcomes from the NHSBT registry were analysed for all patients who received a kidney and one other solid-organ transplant as a child between 2000 and 2021 in the UK. QoL was measured using the PedsQL 3.0 Transplant Module questionnaire. 92 children met the inclusion criteria: heart/heart-lung and kidney (n = 15), liver and kidney (n = 72), pancreas and kidney (n = 4) and multivisceral (n = 1). Results showed excellent patient and graft survival, comparable to single-organ transplants. Allograft survival and rejection were significantly better in patients with combined liver and kidney transplants compared to patients with sequential liver and kidney transplants. QoL was excellent with a mean score of 74%. Key findings included a significant improvement in QoL post-transplant. This is the first study to look at clinical and QoL outcomes in MSOT recipients. The results indicate excellent long-term outcomes. All children born with conditions leading to end-stage disease in multiple solid-organs should be assessed as transplant candidates.

## Graphical Abstract

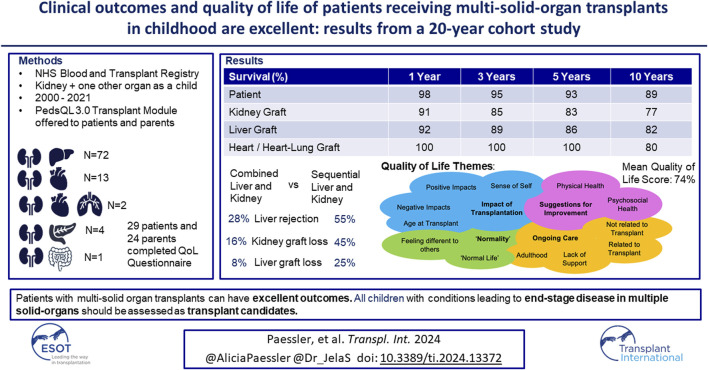

## Introduction

Advances in modern medicine mean that more babies born with complex health conditions survive into childhood, resulting in an increasing number requiring multiple different solid organ transplants (MSOT) (for example, there were only 2 such transplants in the year 2000 in the UK, and 15 years later there was as many as 8 in 1 year [1]). These patients have unique healthcare needs that have not yet sufficiently been explored. Some of these children will have metabolic conditions, and present at an earlier age, bringing challenges in finding size-matched grafts but also in creating adequate vascular anastomosis and achieving abdominal closure for those receiving a graft from an adult. Some children with single-organ transplants go on to need a second organ, (thereby making them MSOT candidates) and for these children sensitisation from their prior transplants can be a significant issue. Caring for a child with one transplanted organ is challenging enough, so it follows that children with MSOT can provide extra challenges for patients and healthcare professionals in both paediatric, and subsequently adult settings.

Despite the increasing number of children requiring MSOT, there is still no large-scale registry data published on their long-term outcomes nor data on how these patients do once they reach adulthood [2–11]. Within the existing limited evidence, there is an ongoing debate as to whether liver and kidney transplants have better outcomes if they are done combined or sequentially. There is a suggested immunological advantage of combined transplants with data showing that the presence of a liver graft from the same donor has an immunological protective effect, with various possible explanations [3, 12–15]. However, some studies report a higher rate of complications and mortality in the first year post-combined liver and kidney transplant (CLKT) when compared to patients undergoing sequential liver and kidney transplants (SLKT), isolated liver transplants (LT) or isolated kidney transplant (KT) [2, 3]. At present, there is no clear consensus favouring one over another.

Furthermore, there is no available data on quality of life (QoL) outcomes for these children. QoL is a crucial aspect of patient’s outcomes; studies have shown increased stress, anxiety and a poorer QoL is correlated with medication non-adherence [16–18]. Medication non-adherence, particularly in adolescents, is a widely reported issue that can lead to more hospitalizations, graft loss and poorer health outcomes [19] so it is key to explore this issue further.

This study is a mixed-methods cohort study that is the first registry study analysis on children with MSOT and to our knowledge, the first one to investigate their QoL. Moreover, it’s one of the largest cohorts of MSOT patients reported and the only one to follow these patients into adulthood. The aim was the following:

To review the long-term clinical outcomes and gain a better understanding of the quality of life of all patients receiving more than one different solid organ transplant during childhood between 2000 and 2021 in the UK.

## Materials and Methods

Those who had received a kidney, plus a minimum of one other solid-organ transplant (either combined or sequentially) in the United Kingdom (UK) before their 18th birthday were eligible for inclusion. The timeframe selected was January 2000 to May 2021. All clinical outcome data was requested and provided by NHS Blood and Transplant (NHSBT) from the UK Transplant registry (UKTR) which includes extensive data on all patients that are listed for transplant, all donors and all transplant outcomes. Informed consent for data collection is obtained from recipients and donor next of kin by NHSBT at the time of listing for transplant/donation. All patient follow-up data was based upon their status on the registry in December 2021. The full details of the UKTR dataset and the variables collected can be viewed online [1]. Throughout this manuscript when abbreviating types of transplants we have described simultaneous transplants as combined (e.g., combined liver and kidney transplant (CLKT)) with multiple different single-organ transplants as sequential transplants (e.g., sequential liver and kidney transplant (SLKT)). We have done this to easier be able to differentiate between the two.

All surviving patients and their families were contacted either via a phone call or at their clinic appointments. They were contacted a maximum of two times and were given an information sheet to allow them to make an informed choice on whether they wanted to consent for the QoL arm of the study. Both patients and parents were asked to complete the PedsQL 3·0 Transplant Module questionnaire either online or on paper [20], with the parent copy of the questionnaire asking parents how they thought their child’s QoL was. The questionnaire assesses how often different aspects of their life are impacted by their transplants, including categories on medication burden, physical appearance, worries, fitting in with their peers etc. Parent copies were given to parents of patients <18 years old and to parents of adult patients where possible. Scores were out of 100 and higher scores indicate a better QoL. Prior to data collection, the authors deemed a score >70 to suggest good QoL to improve understanding of the scores for readers of the manuscript. However, the original tool does not have any validated score cut-offs so this is specific to this study and has not been validated. The questionnaire has age-appropriate versions for 2–4, 5–7, 8–12, 13–18, and >18 years old. Participants were given the opportunity to add any additional comments to the questionnaires about their QoL in a free-text section at the end. Questionnaires were fully anonymised.

IBM Statistical Package for Social Sciences (SPSS) Version 28 [21] was used for all statistical analyses. Patient and graft survival was estimated using Kaplan-Meier analysis and log-rank testing was used to assess comparisons. Multivariable linear regression analysis was carried out where possible. Regression analysis was carried out with the following variables: type of transplant, age at transplant, dialysis status pre-transplant, donor type and underlying disease. P-values, with a threshold of significance of p < 0·05, are displayed as a measure of significance. In the QoL arm of the study, patients were also compared based on age at transplantation by the following groups: <4, 5–7, 8–12, 13–18 years old at time of transplantation. For QoL data, both statistically significant differences and clinically significant differences were reported using Minimally Important Difference values of 0.5 the standard deviation [22]. When data was used for multiple comparisons Bonferroni corrections were implemented. Free-text responses were analysed through thematic analysis [23] to further explore the quantitative data on quality of life in more detail, and add depth to our data on quality of life in MSOT-recipients. Two co-authors read through all the raw data independently to familiarise themselves with the data. Initial codes were identified in the data and then these were grouped into themes. These themes were then compared between the two authors and reviewed and revised. During this process further sub-themes were then identified from the initial codes identified from the raw data.

This study required full ethics approval from the NHS Health Research Authority which was approved under IRAS project ID number 297707 in June 2021. The study was completed in full accordance with ethics approval requirements.

## Results

### Demographics and Background Information

In total, 92 children had MSOT including a kidney, in the UK during the study period. The transplant types, basic demographics and transplant details can be seen in Table 1.

**TABLE 1:** Sex distribution, ethnicities and median ages at time of transplantation organ donor demographics, graft types, HLA match types, and median waiting list times across the different transplant types. Kidney match type is categorised as per NHS Blood and Transplant with favourable including one of the following HLA mismatches: 000, 100, 010 or 110. Letters within the table signify a significant difference (p < 0·05) between variables containing the same letter.

		Total n = 92	Liver and kidney (n = 72)		Heart and kidney (n = 15)		Pancreas and kidney n = 4	Multivisceral n = 1
			Combined n = 53	Sequential n = 19	Combined n = 1	Sequential n = 14		
Sex (%)	Male	52 (57)	33 (62)	9 (47)	0 (0)	9 (64)	1 (25)	0 (0)
	Female	40 (43)	20 (38)	10 (53)	1 (100)	5 (36)	3 (75)	1 (100)
Ethnicity (%)	Asian	14 (15)	9 (17)	3 (16)	0 (0)	2 (14)	0 (0)	0 (0)
	Black	1 (1)	0 (0)	0 (0)	0 (0)	1 (7)	0 (0)	0 (0)
	White	70 (76)	42 (79)	12 (63)	1 (100)	11 (79)	4 (100)	0 (0)
	Other	7 (8)	2 (4)	4 (21)	0 (0)	0 (0)	0 (0)	1 (100)
Median age at time of transplant in years (range)	Kidney	8 (1–17)	7 (1–16)	5 (1–16)	11	13·5 (5–17)	16 (15–17)	12
	Liver	6 (0–19)	7 (1–16)	2 (0–17)	-	-	-	-
	Heart	4 (0–12)	-	-	11	3 (0–12)	-	-
	Heart-Lung	4·5 (4–5)	-	-	-	4.5 (4–5)	-	-
	Pancreas	16 (15–17)	-	-	-	-	16 (15–17)	-
	Multivisceral	12	-	-	-	-	-	12
Kidney Donor Type (%)	DBD	76 (82)	52 (98)	8 (42)	1 (100)	7 (50)	4 (100)	1 (100)
	Living Related	16 (18)	1 (2)	11 (58)	0 (0)	7 (50)	0 (0)	0 (0)
Liver Donor Type (%)	DBD	64 (89)	52 (98)	12 (63)	-	-	-	-
	DCD	1 (1)	0 (0)	1 (6)	-	-	-	-
	Living Related	7 (10)	1 (2)	6 (31)	-	-	-	-
Median Donor Age (range)	Kidney	31 (5–54)	25 (5–49)	34 (12–52)	9	45 (30–54)	27·5 (14–45)	24
	Liver	24·5 (5–57)	25 (5–49)	28 (6–57)	-	-	-	-
	Heart	9 (0–39)	-	-	9	7 (0–39)	-	-
	Heart-Lung	4 (2–6)	-	-	-	4 (2–6)	-	-
	Pancreas	27·5 (14–45)	-	-	-	-	27·5 (14–45)	-
	Multivisceral	24	-	-	-	-	-	24
Kidney Match Type (%)	Favourable	20 (22)	4 (8)	9 (47)	0 (0)	6 (43)	1 (25)	0 (0)
	Non-Favourable	72 (78)	49 (92)	10 (53)	1 (100)	8 (57)	3 (75)	1 (100)
Liver Graft Type (%)	Reduced	14 (19)	6 (12)	8 (42)	-	-	-	-
	Split	38 (53)	32 (60)	6 (32)	-	-	-	-
	Whole	20 (28)	15 (28)	5 (26)	-	-	-	-
Pre-emptive Kidney Transplant (%)	Yes	59 (64)	27 (51) b,c	2 (10) b	1 (100)	2 (15) c	0 (0)	1 (100)
	No	33 (36)	25 (49)	17 (90)	0 (0)	12 (85)	4 (100)	0 (0)
Median Days Spent on the Waiting List (range)	Kidney	210 (13–2,287)	109 (13–1,430) a	504 (39–1,552) a	Not Reported	145 (30–2,287)	219 (175–2,287)	178
	Liver	Not reported	109 (13–1,430)	Not Reported	-	-	-	-
	Heart	111 (1–347)	-	-	Not Reported	111 (1–347)	-	-
	Heart-Lung	510 (99–921)	-	-	-	510 (99–921)	-	-
	Pancreas	219 (175–2,287)	-	-	-	-	219 (175–2,287)	-
	Multivisceral	178	-	-	-	-	-	178

DBD, Donation after brainstem death; DCD, donation after circulatory determination of death.

Underlying medical conditions can be seen in Supplementary Material S1.

### Clinical Outcomes

The median follow-up times post-transplant for the different transplant types can be seen in Table 2. In the SLKT group there was a median of 2 years between liver and kidney transplants, in the H/HLKT group there was a median of 9.5 years between the heart/heart-lung and kidney transplant.

**TABLE 2:** Median follow up times post-transplant across the different transplant types.

	Total (n = 92)	Liver and kidney (n = 72)		Heart and kidney (n = 15)		Pancreas and kidney (n = 4)	Multivisceral (n = 1)
		CLKT (n = 53)	SLKT (n = 19)	CHKT (n = 1)	SHKT (n = 14)		
Median Follow up Time (years) Post-Kidney Transplant (Range)	8·4 (0–22·5)	8·8 (0·2–22·5)	7·3 (0–18·6)	9·8	7·6 (0·9–15·6)	6·5 (0–16·4)	1·0
Median Follow up Time (years) Post-Liver Transplant (Range)	8·7 (0·1–26·9)	8·8 (0·2–22·5)	7·3 (0·1–26·9)	-	-	-	-
Median Follow up Time (years) Post-Heart Transplant (Range)	14·6 (3·0–29·6)	-	-	9·8	14·2 (3·0–29·6)	-	-
Median Follow up Time (years) Post-Heart-Lung Transplant (Range)	23·2 (21·9–24·4)	-	-	-	23.2 (21.9–24.4)	-	-
Median Follow up Time (years) Post-Pancreas Transplant (Range)	6·5 (0–16·4)	-	-	-	-	6·5 (0–16·4)	-
Median Follow up Time (years) Post-Multivisceral Transplant (Range)	1·0	-	-	-	-	-	1·0

CLKT, Combined Liver and Kidney Transplant; SLKT, Sequential Liver and Kidney Transplant; CHKT, Combined Heart and Kidney Transplant; SHKT, Sequential Heart and Kidney Transplant.

### Clinical Outcomes: Allograft Function

Serum creatinine levels remained stable at 3 months, one and 5 years post-KT (mean 82 ± 54, 82 ± 69, and 93 ± 44 μmol/L respectively). There was no statistically significant difference between the transplant types. However, when comparing those with kidney and liver transplants, the serum creatinine was significantly lower at 5 years post-transplant for patients in whom both organs had come from the same donor (i.e., combined transplant from one deceased donor or sequential transplants from the same living donor) (82 μmol/L vs. 104 μmol/L, p = 0·02).

For patients undergoing H/HLT, prior to transplant the majority (66% n = 6) were classified as New York Heart Association (NYHA) Heart Failure Class IV [24]. At 10 years post-transplant, 100% of patients were classified as NYHA Heart Failure Class I [24]. At 10 years post-H/HLT, patients had an average of seven hospital admissions post-transplant.

Patients had a median of five and four (CLKT and SLKT respectively) hospital admissions in the first 5 years post-transplant and their lifestyle activity score significantly improved post-transplant, where pre-transplant only 5% of patients were able to carry out normal activity without restriction and post-transplant this increased to 79% (p < 0·01).

### Clinical Outcomes: Rejection

In the first 5 years post-transplant, 7 patients (7%) had experienced episodes of kidney rejection. These occurred in 9% (n = 3) of CLKT patients, 9% (n = 1) of SLKT patients and 20% (n = 3) of heart/heart-lung and kidney transplant (H/HLKT) patients. 14 patients (19%) had experienced episodes of liver rejection, which was significantly higher in SLKT patients (n = 5, 36% vs. n = 9, 10%, p = 0.01) in the first 5 years post-transplant. 4 patients (27%) had experienced episodes of heart rejection at 5 years post-transplant. Multivariable analysis did not find any variables that significantly impacted episodes of any graft rejection.

### Clinical Outcomes: Graft Survival

Patients with grafts from the same donor were less likely to lose their kidney grafts than patients with grafts from different donors (p < 0·01). Similarly, CLKT patients were less likely to lose their liver graft than SLKT patients (p < 0·01). The causes of graft loss can be seen in Supplementary Material S1.

**FIGURE 1:**
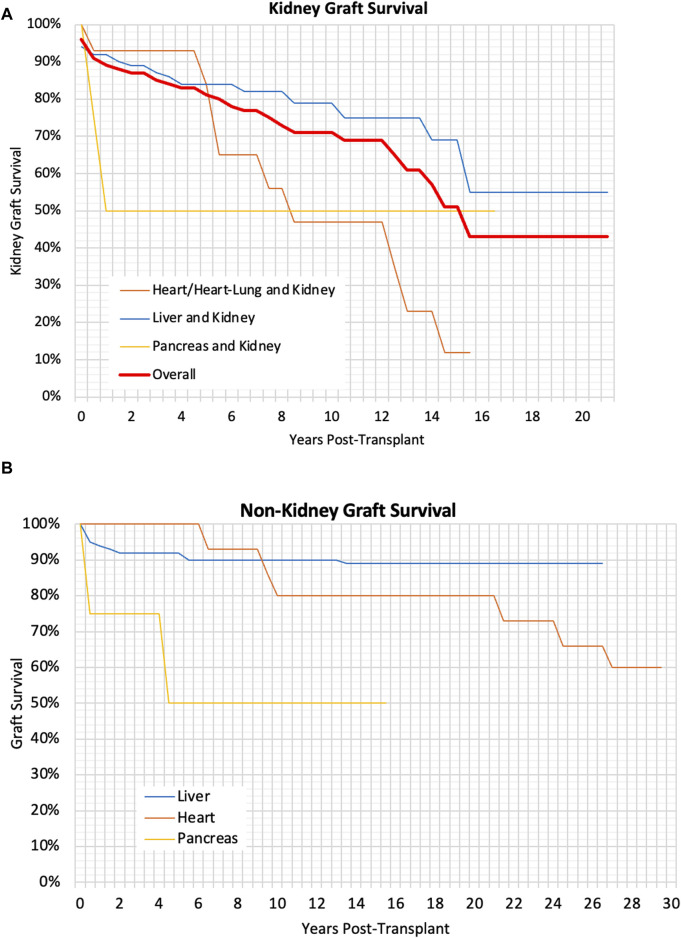
**(A)** Kaplan Meier Graph showing the kidney graft survival for different types of transplants, p < 0.01, **(B)** Kaplan Meier Graph showing the graft survival of liver, heart and pancreas grafts, p < 0.01.

Liver graft survival was found to be significantly better in CLKT patients compared to SLKT patients (p < 0·01). There was no significant difference in kidney graft survival between the CLKT and SLKT group, however kidney graft survival was significantly better in liver and kidney patients compared to heart/heart-lung and kidney patients (p < 0·01). However once death-censored this difference was no longer significant. Multivariable analysis did not find any other variables which impacted graft survival. There was no significant difference in graft survival for children transplanted at different ages (p = 0.55). Graft survival can be seen in Table 3 and Kaplan-Meier curves for graft survival can be seen in Figures 1A, B.

**TABLE 3:** Graft and death-censored graft survival at 1, 3, 5, and 10 years post-transplant for different transplant types. Letters within the table signify a significant difference (p < 0·05) between variables containing the same letter.

		Total (n = 92)	Liver and kidney (n = 72)		Heart/Heart-lung and kidney (n = 15)	Pancreas and kidney (n = 4)	Multi-visceral (n = 1)
			CLKT (n = 53)	SLKT (n = 19)			
Kidney Graft Survival (%)	1 year	91	91	94	93	50	100
	3 years	85	87	89	93	50	0
	5 years	83	85	83	86	50	0
	10 years	77	81	83	47	50	0
Death Censored Kidney Graft Survival (%)	1 year	91	92	94	93	50	100
	3 years	88	91	89	93	50	0
	5 years	86	89	89	86	50	0
	10 years	82	85	89	62	50	0
Liver Graft Survival (%)	1 year	92	96 a	79 a	-	-	100
	3 years	89	92 b	79 b	-	-	0
	5 years	89	92 c	79 c	-	-	0
	10 years	86	92 d	68 d	-	-	0
Death Censored Liver Graft Survival (%)	1 year	93	98 e	79 e	-	-	100
	3 years	92	96 f	79 f	-	-	0
	5 years	92	96 g	79 g	-	-	0
	10 years	89	96 h	68 h	-	-	0
Heart/ Heart-Lung Graft Survival (%)	1 year	100	-	-	100	-	-
	3 years	100	-	-	100	-	-
	5 years	100	-	-	100	-	-
	10 years	80	-	-	80	-	-
Death Censored Heart/ Heart-Lung Graft Survival (%)	1 year	100	-	-	100	-	-
	3 years	100	-	-	100	-	-
	5 years	100	-	-	100	-	-
	10 years	87	-	-	87	-	-
Pancreas Graft Survival (%)	1 year	75	-	-	-	75	-
	3 years	75	-	-	-	75	-
	5 years	50	-	-	-	50	-
	10 years	50	-	-	-	50	-
Multi-visceral Graft Survival (%)	1 year	100	-	-	-	-	100
	3 years	0	-	-	-	-	0
	5 years	0	-	-	-	-	0
	10 years	0	-	-	-	-	0

CLKT = Combined Liver and Kidney Transplant, SLKT = Sequential Liver and Kidney Transplant.

### Clinical Outcomes: Patient Survival

Overall, 14 patients died during the study period, this was not significantly different between the transplant types. Causes of death can be seen in Supplementary Material S1.

**FIGURE 2:**
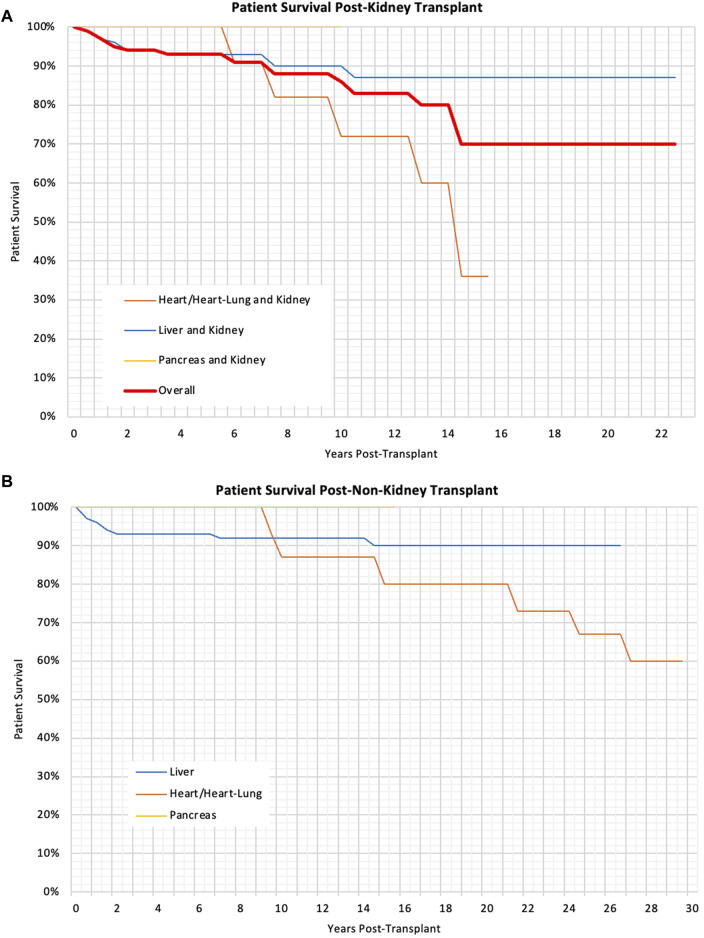
**(A)** Kaplan Meier Graph showing the patient survival post-kidney transplant for different types of transplants, p < 0.01 **(B)** Kaplan Meier Graph showing the patient survival following liver, heart and pancreas, p = 0.03.

There was no significant difference in the patient Kaplan-Meier survival rates and multivariable analysis found no variables that significantly impacted these. There was no significant difference in patient survival for children transplanted at different ages (p = 0.55). The patient survival can be seen in Table 4 and the Kaplan-Meier survival curves can be seen in Figures 2A, B.

**TABLE 4:** Patient survival at 1, 3, 5, and 10 years post-transplant for different transplant types.

		Total (n = 92)	Liver and kidney (n = 72)		Heart/Heart-lung and kidney (n = 15)	Pancreas and kidney (n = 4)	Multi-visceral (n = 1)
			CLKT (n = 53)	SLKT (n = 19)			
Patient Survival Post-Kidney Transplant (%)	1 year	98	96	100	100	100	100
	3 years	95	93	100	100	100	0
	5 years	93	93	93	100	100	0
	10 years	89	93	85	71	100	0
Patient Survival Post-Liver Transplant (%)	1 year	96	96	95	-	-	100
	3 years	93	93	95	-	-	0
	5 years	93	93	95	-	-	0
	10 years	92	93	90	-	-	0
Patient Survival Post-Heart/Heart-Lung Transplant (%)	1 year	100	-	-	100	-	-
	3 years	100	-	-	100	-	-
	5 years	100	-	-	100	-	-
	10 years	87	-	-	87	-	-
Patient Survival Post-Pancreas Transplant (%)	1 year	100	-	-	-	100	-
	3 years	100	-	-	-	100	-
	5 years	100	-	-	-	100	-
	10 years	100	-	-	-	100	-
Patient Survival Post-Multivisceral Transplant (%)	1 year	100	-	-	-	-	100
	3 years	0	-	-	-	-	0
	5 years	0	-	-	-	-	0
	10 years	0	-	-	-	-	0

CLKT, Combined Liver and Kidney Transplant; SLKT, Sequential Liver and Kidney Transplant.

### Quality of Life

Out of the 78 surviving patients, 46 were identified through their local transplant centre. Of these, we were able to contact and consent 37 to the QoL arm of the study. Finally, Thirty-one families returned their questionnaires which included 29 patients and 24 parents. The distribution across the different transplant types was representative of the number of patients of each transplant type in the clinical arm of the study (20 CLKT patients, 8 SLKT patients, 8 HKT patients). The median age of the patients at the time of participation was 16 years (ranging between 4 and 32 years old). 21 were still under the age of 18 and 10 had become adults. The median time since transplantation was 7 years (range 0·3–17·5 years). Validity of the questionnaire results were analysed using Cronbach’s coefficient alpha and can be seen in Supplementary Material S1.

**FIGURE 3:**
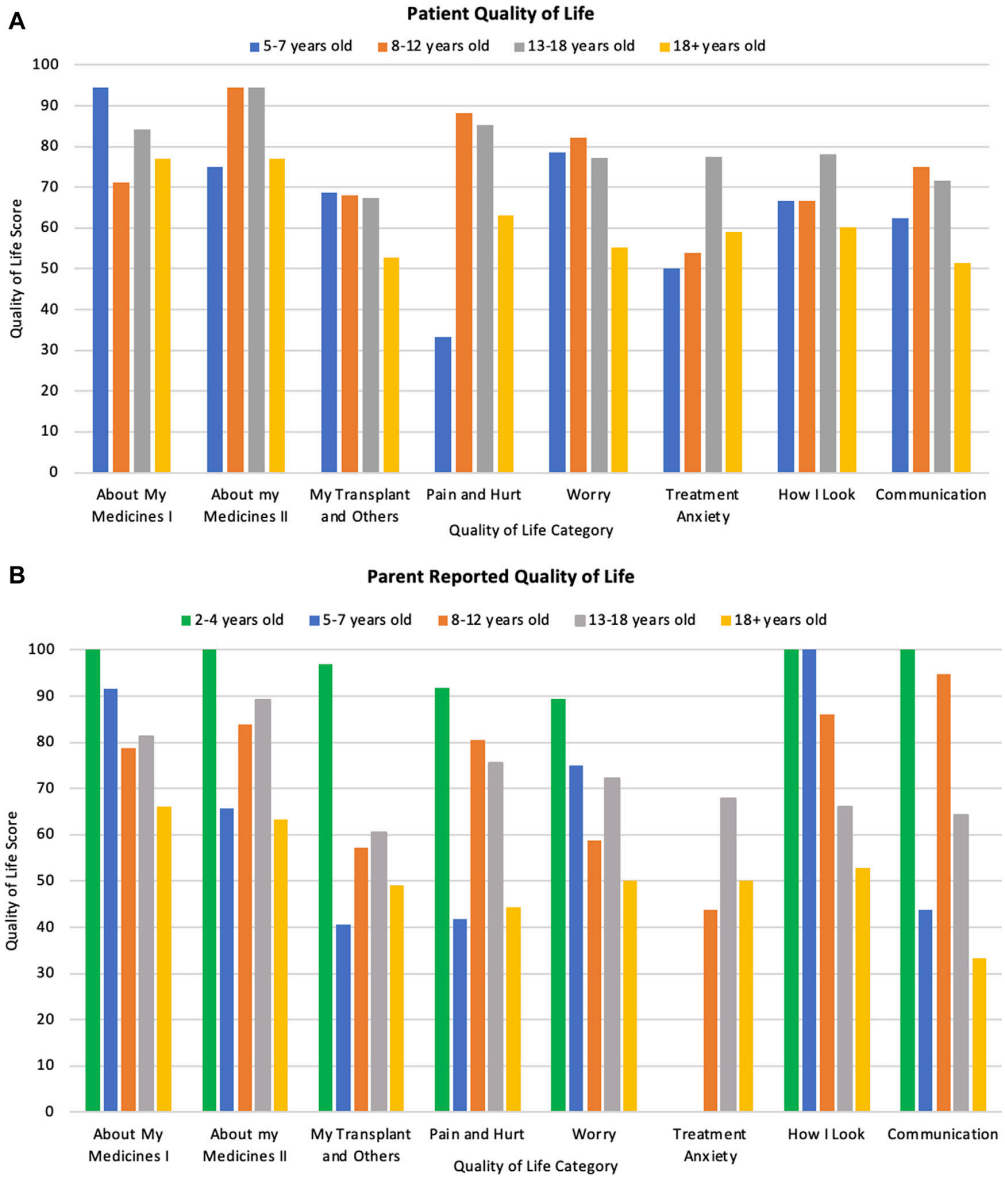
**(A)** Patient reported quality of life scores across different ages and categories at time of response. **(B)** Parent reported scores of their child’s quality of life across different patient ages and categories.

Overall patient and parent scores can be seen in Table 5 as well as by the different age categories at the time of response in Figures 3A, B. Minimally important difference values for each category, that are used to determine clinical significance of changes in each category are also listed in Table 5. Overall, patient self-reports had higher QoL scores than parent-proxy QoL scores, however this was not statistically significant. (p = 0.44).

**TABLE 5:** Patient and Parent reported quality of life scores across all categories with 95% Confidence Intervals and minimally important difference values.

	Patient mean	Patient 95% CI	Minimally important difference (0.5 SD)	Parent mean	Parent 95% CI	Minimally important difference (0.5 SD)
About My Medicines I	79·9	73·6–86·2	9.0	79·9	77·9–81·9	9.0
About My Medicines II	88·2	82·6–93·7	7.9	84·1	76·7–91·5	9.3
My Transplant and Others	62·8	55·2–70·5	10.9	58·9	48·8–69	12.7
Pain and Hurt	76·8	68·3–85·3	12.2	72·2	62·2–82·2	12.5
Worry	71·1	63·7–78·4	10.5	66·9	55·2–78·6	14.7
Treatment Anxiety	66·3	55·3–77·3	15.6	53·9	37·4–70·4	20.6
How I Look	69·9	61·9–78	11.4	72·2	60·5–84	14.7
Communication	65·4	54·3–76·5	15.7	68·8	54·8–82·7	17.4
Total	**73·7**	**68**·**2–79**·**2**	**7.8**	**70**·**1**	**62**·**7–77**·**4**	**9.2**

SD, standard deviation.

Bold values represent the overall results across the whole questionnaire.

Patients who were transplanted at a younger age had a significantly better QoL (both statistically significant and clinically significant) across every category (p < 0·01) when compared to those transplanted at an older age (Total mean QoL score of 79.5, 78.6, 73.4, and 56.0 for patients transplanted at age <4, 5–7, 8–12, and 13–18 respectively). Patient QoL significantly decreased with age in relation to medication burden, pain, worry and communication (p = 0·02, 0·02, 0·01, and 0·03 respectively) as displayed in Figures 3A, B. However, there was no difference in overall QoL between the different transplant types (p = 0.94) nor by time since transplantation (p = 0.39). These differences were also apparent when testing for clinical significance.

**FIGURE 4:**
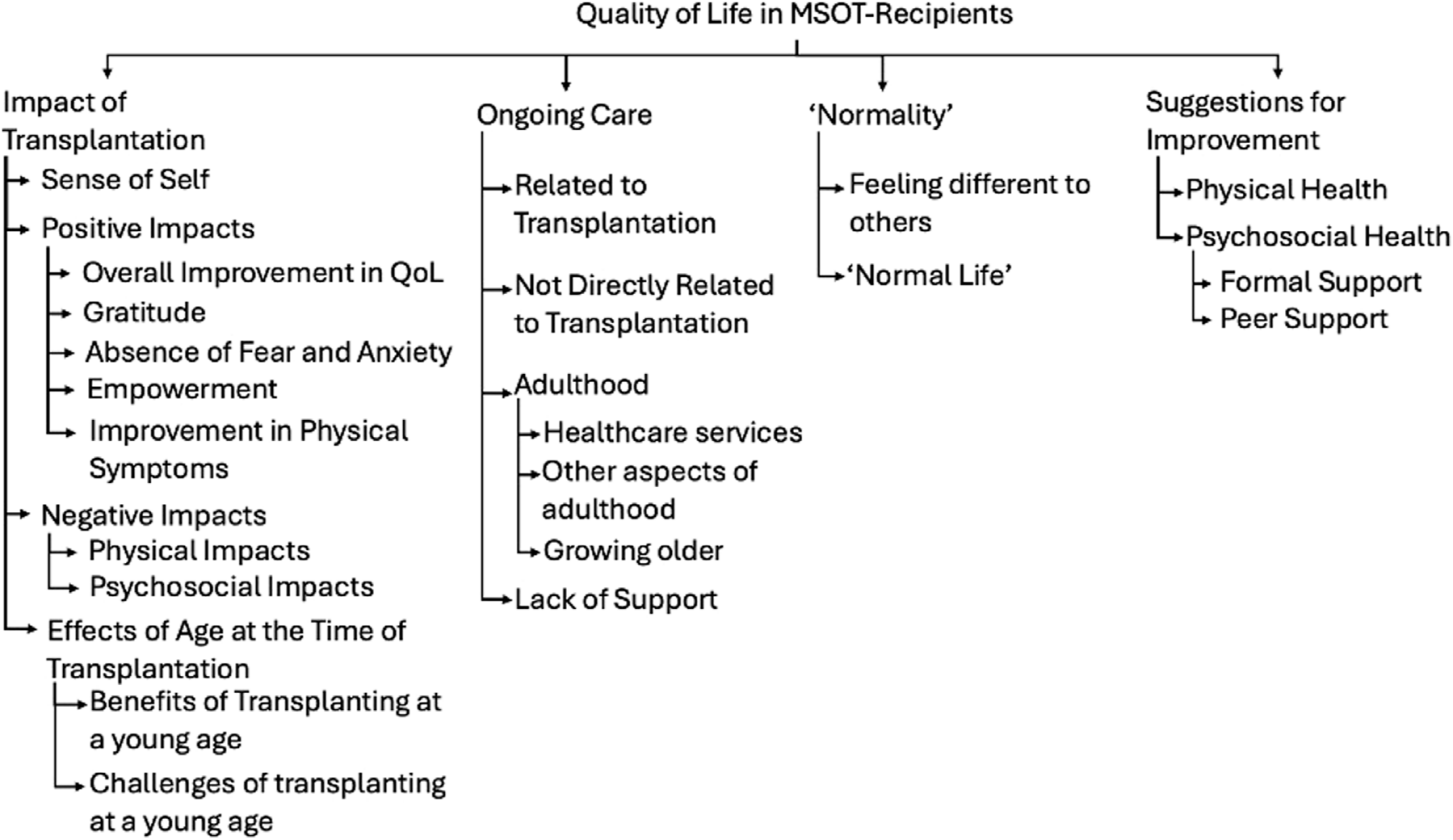
Themes and Sub-themes identified in thematic analysis of free-text responses in quality of life questionnaire.

These trends were explored further with thematic analysis of the additional comments left by patients and parents. The analysis produced four overarching themes with 12 sub-themes. Themes and sub-themes can be seen in Figure 4 and the data including participant quotes can be seen in Supplementary Material S2.

The first main theme explored the impact of transplantation. Receiving a MSOT had a significant impact of patients’ sense of self’, with some describing themselves as “a completely different person” (Patient 6) pre- and post-transplant. Many positive impacts of transplantation were also discussed, with participants saying that their “Quality of life improved tremendously” (Parent 10). Sub-themes included feelings of gratitude, absence of fear or anxiety, feelings of empowerment and an improvement in physical symptoms. Naturally, negative impacts of transplantation were also explored which included physical consequences such as vulnerability to infections, as well as the impact on mental health. The age at transplantation was often referred to, with some participants feeling that being transplanted at a younger age was beneficial either due to the lack of memories of the transplant itself or because it was always a part of normal life whilst they were growing up. Conversely, some commented that being transplanted young was more challenging with one parent saying “the trauma of becoming so poorly and to need so much intervention at such a young age is underestimated” (Parent 31).

The second theme was about ‘Normality’ and what our patient’s experience of “normality was. Naturally, the idea of “normalcy” is very abstract and will mean something different to every person and is something that many adolescents seek irrespective of underlying health conditions. Healthcare professionals should be careful not to try to define “normality” and should not reinforce “normalcy” as a binary concept that separates children with underlying health conditions from others. However, patients and parents did widely report their experience with seeking “normality” and how this changed post-transplant. Feeling different to others was challenging for many patients, however was seen as a positive thing in others. This partially related to physical differences, but also through missing out on life experiences during childhood due to illness. There was also some reference to a “normal life” and what that looked like post-transplant, with some stating that their life had become much more “normal” post-transplant.

The third theme related to ongoing care post-transplant. The challenges of transitioning into adulthood was deemed especially important. Participants described that the transition to adult services was “very difficult” (Parent 6), with less perceived support than the paediatric setting. They also described concerns about equal employment opportunities in the workplace. Concerns about the future appeared to become more prevalent as MSOT recipients grew older, with concerns about the implications of their transplant status. Furthermore, there was a number of comments made about the lack of psychosocial support post-transplant and how this remained a key issue impacting QoL.

Finally, there were also many suggestions for how transplant services could be improved, not only with further support for physical symptoms, but also more support for mental health. There were suggestions for both formal support and the potential value of peer support for the psychosocial health of MSOT recipients.

## Discussion

The results from this study have shown that patients who receive MSOT during childhood can have excellent long-term physical and quality of life outcomes with the right support from the multi-disciplinary team.

Overall, the graft survival, across all types of patients, particularly when death censored, is comparable to other studies looking at CLKT/SLKT and HKT outcomes [2, 5, 9, 25] and is similar if not better, than after single organ transplants as per the United Network for Organ Sharing (UNOS), European Society of Paediatric Nephrology/ European Renal Association/ European Dialysis and Transplantation Association (ESPN/ERA/EDTA), European Liver Transplant Registry (ELTR) and International Thoracic Organ Transplant Registry (ITOTR) registry data [8, 26–28]. For example, our kidney graft survival was 91% and 83% at 1 and 5 years, and the UNOS single-kidney graft survival was 95%–97% and 78%–88% across the same time periods. For liver transplants, our graft survival was 92% and 89% at 1 and 5 years, while UNOS single-liver graft survival was 86%–92% and 79%–87%. Our heart graft survival was 100% both at 1 and 5 years, compared to 87%–96% and 75%–84% from UNOS data for single-heart graft survival [28]. One possible reason for the excellent liver graft outcomes is that at least 40% of these children did not have end stage liver disease (ESLD) prior to transplant, but the liver was replaced for other reasons, such as replacing deficient enzyme activity in metabolic conditions. Existing data suggests that children with ESLD have poorer outcomes post-transplant so this may be a contributing factor to the positive outcomes in this study [29]. Another possible explanation for the improved graft survival in MSOT recipients is that these patients may have extra follow-ups with more than one specialist team (i.e., both with hepatologists/cardiologists and nephrologists) than children with isolated single-organ transplants. Therefore, any complications or findings that may impact graft survival may be picked up quicker [30]. It is also likely that for these reasons, our patient survival was excellent and equally comparable to single-organ transplant recipients. Renal insufficiency is often cited as a relative contraindication to heart transplantation [31], but our data shows that these patients can still have excellent outcomes and so MSOT should still be carefully considered [11].

In terms of liver and kidney patients, our study suggests that CLKT may be a better option – with better outcomes for both the liver and kidney grafts. Furthermore, patients waited longer for their deceased donor KT in the SLKT group than in the CLKT group, and so were less likely to undergo pre-emptive transplants. This is possibly because multi-organ transplants are prioritised in the UK organ allocation process (thereby favouring the CLKT group), or may be because children receiving a KT after a liver transplant may be sensitised from their previous transplant [32, 33]. Current evidence highlights the improved outcomes after pre-emptive transplantation [34], so this is an important factor to consider when deciding to opt for CLKT or SLKT. We also did not find a difference in the rate of complications or mortality in the first-year post-transplant between the two groups. This is reassuring and suggests that an increased potential for complications or higher mortality in the first year post-transplant should not be the sole reason not to list a child for a CLKT if they are otherwise suitable.

A significant strength in this study is the use of a validated, transplant-specific to collect mixed methods data on quality of life; something which is relatively under investigated and therefore poorly quantified in all areas of paediatric transplantation.

One of our main findings is that children who are transplanted at a younger age have a significantly better QoL despite the fact that age at transplantation did not affect clinical outcomes. Our qualitative data indicates that this may be due to a lack of memory of the transplant itself, or of life before it was deemed a necessity. These children are likely to grow up with the identity of being a transplant recipient already embedded in their sense of self. Conversely older children may recall a time before they were unwell and must grow accustomed to their transplant recipient status from a place of prior ‘normality’. Similarly, a study looking at QoL in paediatric LT recipients found older age at transplantation to be a predictor for poorer QoL [35]. We also found that QoL was worse in older patients, particularly with respects to medication burden and worries. Patients described that as they became older and more mature, they thought more about the implications of their health conditions, and they noticed greater differences between themselves and others. Increased worry and anxiety around the future may have significant implications on mental health and also on medication adherence. Although adherence was not formally investigated in this study, the About My Medicines I section of the questionnaire does explore patients’ medication burden which showed that that adolescents struggled more with their medication burden than younger patients, putting them at higher risk of non-adherence. A key contributing factor to non-adherence is transition to adult services [36] which can be challenging, and some of our patients reported struggling with this. While our data shows better QoL in those transplanted at an earlier age, the clinical application of this finding is somewhat limited by our relatively small sample size. However, clinicians should be aware of this finding when assessing their patients who were transplanted at an older age.

The need for greater mental health support was very clearly identified within this study. Whilst it is well known that mental health issues are increasing in children for numerous reasons [37, 38], our cohort poses unique mental health needs that require a specialized and multidisciplinary approach, with equal focus on these and physical outcomes. Whilst participants did indicate the need for formal mental health support, peer support was also raised as an appropriate alternative. Such suggestions should be considered as part of standard care for MSOT recipients, particularly for adolescents as part of transition programs to protect the most vulnerable cohort of patients.

Although this study is limited by its sample size and the homogenous nature of the participants, it remains one of the largest cohort studies of MSOT to have been performed. However, all clinical data used has come from the UKTR, which is a reliable source that contains clinical data on every eligible patient, who was subsequently included in the analysis. Furthermore, this cohort has a long follow-up period and includes a large number of children reaching adulthood, and therefore provides high quality data to assess long-term outcomes. One of the possible limitations with our QoL data is that only <40% of patients and only surviving patients and families were surveyed. It is possible that patients who unfortunately died post-transplant or patients that did not participate in the QoL arm of the study had a very different QoL experience when compared to those who were able to participate.

Whilst it is encouraging to see that patients undergoing MSOT in childhood can have equally good outcomes as children requiring a single organ, it is important to note that single organ transplants are not a suitable option for these patients. A more worthwhile comparison would be to compare this cohort with the outcomes of patients who require MSOT but do not undergo transplantation, however such data is not readily available. We would also encourage prospective research in this field, starting prior to transplantation with participants completing annual QoL questionnaires both pre-and post-transplant to further identify trends and new issues in QoL.

## Conclusion

This is the largest study presenting data on the outcomes of recipients of MSOT followed up over two decades. It is the first to look at all organ combinations and quality of life outcomes. It demonstrates that patients undergoing MSOT in childhood have excellent outcomes in terms of graft function, graft and patient survival, and QoL. These outcomes are all comparable to those undergoing single-organ transplants in the literature. Both liver and kidney graft survival and rates of rejection were found to be better in patients undergoing CLKT when compared to SLKT in this cohort of patients. QoL can be excellent with the support of the multi-disciplinary team which is crucial throughout patients’ transplant journeys. The evidence in this study is supportive of children with the need for multiple organ replacement being considered for multi-organ transplantation.

## Data Availability

The original contributions presented in the study are included in the article/Supplementary Material, further inquiries can be directed to the corresponding author.

## Abbreviations

CLKT, Combined Liver and Kidney Transplant; ESLD, End Stage Liver Disease; EDTA, European Dialysis and Transplantation Association; ELTR, European Liver Transplant Registry; ERA, European Renal Association; ESPN, European Society for Paediatric Nephrology; H/HLKT, Heart/Heart-lung and kidney transplant; SPSS, IBM Statistical Package for Social Sciences; ITOTR, International Thoracic Organ Transplant Registry; KT, Kidney Transplant; LT, Liver Transplant; MSOT, Multi-solid-organ-transplant; NHSBT, National Health Service Blood and Transplant; NYHA, New York Heart Association; QoL, Quality of Life; SLKT, Sequential Liver and Kidney Transplant; UK, United Kingdom; UKTRUnited Kingdom Transplant Registry; UNOS, United Network for Organ Sharing.

## References

[1] Transplant NBa. Organ Donation and Transplantation Clinical (2024).

[2] Calinescu AM Wildhaber BE Poncet A Toso C McLin VA. Outcomes of Combined Liver–Kidney Transplantation in Children: Analysis of the Scientific Registry of Transplant Recipients. Am J Transplant (2014) 14(12):2861–8. 10.1111/ajt.12935 25274400

[3] Sutherland SM Alexander SR Sarwal MM Berquist WE Concepcion W. Combined Liver-Kidney Transplantation in Children: Indications and Outcome. Pediatr Transpl (2008) 12(8):835–46. 10.1111/j.1399-3046.2008.01041.x 19000066

[4] Perera MT Silva MA Sharif K McKiernan PJ Kelly DA Lloyd C Improved Outcomes of Combined Liver and Kidney Transplants in Small Children (<15 Kg). Transplantation (2009) 88(5):711–5. 10.1097/TP.0b013e3181b29f0c 19741470

[5] Loos S Kemper MJ Schmaeschke K Herden U Fischer L Hoppe B Long-Term Outcome After Combined or Sequential Liver and Kidney Transplantation in Children WITH Infantile and Juvenile Primary Hyperoxaluria TYPE 1. Front Pediatr (2023) 11:1157215. 10.3389/fped.2023.1157215 37009285 PMC10064088

[6] Brinkert F Lehnhardt A Montoya C Helmke K Schaefer H Fischer L Combined Liver-Kidney Transplantation for Children WITH Autosomal Recessive Polycystic Kidney Disease (ARPKD): Indication and Outcome. Transpl Int (2013) 26(6):640–50. 10.1111/tri.12098 23582048

[7] Büscher R Büscher AK Cetiner M Treckmann JW Paul A Vester U Combined Liver and Kidney Transplantation and Kidney After Liver Transplantation in Children: Indication, Postoperative Outcome, and Long-Term Results. Pediatr Transpl (2015) 19(8):858–65. 10.1111/petr.12595 26341656

[8] Rossano JW Cherikh WS Chambers DC Goldfarb S Hayes D Khush KK The International Thoracic Organ Transplant Registry of the International Society for Heart and LUNG Transplantation: Twenty-First Pediatric Heart Transplantation Report—2018; Focus Theme: Multiorgan Transplantation. The J Heart Lung Transplant (2018) 37(10):1184–95. 10.1016/j.healun.2018.07.018 30293614

[9] Weng PL Alejos JC Halnon N Zhang Q Reed EF Tsai Chambers E. Long-Term Outcomes of Simultaneous Heart and Kidney Transplantation in Pediatric Recipients. Pediatr Transpl (2017) 21(7). 10.1111/petr.13023 PMC5638697 28727227

[10] Choudhry S Denfield SW Dharnidharka VR Wang Y Price JF Cabrera AG Simultaneous Pediatric Heart-Kidney Transplant Outcomes in the US: A 25 YEAR National Cohort Study. The J Heart Lung Transplant (2019) 38(4):S114. 10.1016/j.healun.2019.01.269 34585490

[11] Dani A Price N Thangappan K Ryan TD Hooper DK Cooper DS Heart-Kidney Listing Is Better THAN Isolated Heart Listing for Pediatric Heart Transplant Candidates WITH Significant Renal Insufficiency. J Thorac Cardiovasc Surg (2022) 164(6):2019–31. 10.1016/j.jtcvs.2021.10.082 35331555 PMC9433468

[12] Chava SP Singh B Pal S Dhawan A Heaton ND. Indications for Combined Liver and Kidney Transplantation in Children. Pediatr Transpl (2009) 13(6):661–9. 10.1111/j.1399-3046.2008.01046.x 19566856

[13] Grenda R Kaliciński P. Combined and Sequential Liver–Kidney Transplantation in Children. Pediatr Nephrol (2018) 33(12):2227–37. 10.1007/s00467-017-3880-4 29322327 PMC6208698

[14] Kitajima K Ogawa Y Miki K Kai K Sannomiya A Iwadoh K Longterm Renal Allograft Survival After Sequential Liver-Kidney Transplantation FROM a Single Living Donor. Liver Transplant (2017) 23(3):315–23. 10.1002/lt.24676 27862900

[15] Chandak P Kostakis ID Charalambides MM Gogalniceanu P Papadakis G Stojanovic J Kidney Graft Outcomes Following Kidney Versus Simultaneous Liver-Kidney Transplantation –an UNOS Database Analysis Pediatric Transplantation (2023). p. 18.

[16] Compas BE Jaser SS Dunn MJ Rodriguez EM. Coping WITH Chronic Illness in Childhood and Adolescence. Annu Rev Clin Psychol (2012) 8:455–80. 10.1146/annurev-clinpsy-032511-143108 22224836 PMC3319320

[17] Chantzaras A Yfantopoulos J. Association Between Medication Adherence and Health-Related Quality of LIFE of Patients WITH Diabetes. Hormones (Athens) (2022) 21(4):691–705. 10.1007/s42000-022-00400-y 36219341 PMC9552716

[18] Shafiekhani M Shahabinezhad F Tavakoli Z Tarakmeh T Haem E Sari N Quality of LIFE Associated WITH Immunosuppressant Treatment Adherence in Liver Transplant Recipients: A Cross-Sectional Study. Front Pharmacol (2023) 14:1051350. 10.3389/fphar.2023.1051350 36909168 PMC9998979

[19] Simons LE Blount RL. Identifying Barriers to Medication Adherence in Adolescent Transplant Recipients. J Pediatr Psychol (2007) 32(7):831–44. 10.1093/jpepsy/jsm030 17522111

[20] Weissberg-Benchell J Zielinski TE Rodgers S Greenley RN Askenazi D Goldstein SL Pediatric Health-Related Quality of LIFE: Feasibility, Reliability and Validity of the PedsQL™ Transplant Module. Am J Transplant (2010) 10(7):1677–85. 10.1111/j.1600-6143.2010.03149.x 20642689

[21] IBM. Statistical Package for Social Sciences. 28.0.1.0. Chicago, USA: IBM (2022).

[22] Norman GR Sloan JA Wyrwich KW. Interpretation of Changes in Health-Related Quality of LIFE: The Remarkable Universality of HALF a Standard Deviation. Med Care (2003) 41(5):582–92. 10.1097/01.MLR.0000062554.74615.4C 12719681

[23] Braun V Clarke V. Using Thematic Analysis in Psychology. Qual Res Psychol (2006) 3(2):77–101. 10.1191/1478088706qp063oa

[24] White PD Myers MM. The Classification of Cardiac Diagnosis. Jama (1921) 77(18):1414–5. 10.1001/jama.1921.02630440034013

[25] Ranawaka R Lloyd C McKiernan PJ Hulton SA Sharif K Milford DV. Combined Liver and Kidney Transplantation in Children: Analysis of Renal Graft Outcome. Pediatr Nephrol (2016) 31(9):1539–43. 10.1007/s00467-016-3396-3 27105881

[26] Bonthuis M Vidal E Bjerre A Aydoğ Ö Baiko S Garneata L Ten-Year Trends in Epidemiology and Outcomes of Pediatric Kidney Replacement Therapy in Europe: DATA FROM the ESPN/ERA-EDTA Registry. Pediatr Nephrol (2021) 36(8):2337–48. 10.1007/s00467-021-04928-w 33483800 PMC8260419

[27] Baumann U Karam V Adam R Fondevila C Dhawan A Sokal E Prognosis of Children Undergoing Liver Transplantation: A 30-YEAR European Study. Pediatrics (2022) 150(4):e2022057424. 10.1542/peds.2022-057424 36111446

[28] Sharing UNoO. National Data (2015).

[29] Kim WR Lake JR Smith JM Skeans MA Schladt DP Edwards EB OPTN/SRTR 2015 Annual DATA Report: Liver. Am J Transplant (2017) 17(S1):174–251. 10.1111/ajt.14126 28052604

[30] Rawal N Yazigi N. Pediatric Liver Transplantation. Pediatr Clin North America (2017) 64(3):677–84. 10.1016/j.pcl.2017.02.003 28502445

[31] Schweiger M Stiasny B Dave H Cavigelli-Brunner A Balmer C Kretschmar O Pediatric Heart Transplantation. J Thorac Dis (2015) 7(3):552–9. 10.3978/j.issn.2072-1439.2015.01.38 25922739 PMC4387410

[32] NHS. Introduction to Patient Selection and Organ Allocation Policies (2017).

[33] Mamode N Bestard O Claas F Furian L Griffin S Legendre C European Guideline for the Management of Kidney Transplant Patients WITH HLA Antibodies: By the European Society for Organ Transplantation Working Group. Transpl Int (2022) 35:10511. 10.3389/ti.2022.10511 36033645 PMC9399356

[34] Kallab S Bassil N Esposito L Cardeau-Desangles I Rostaing L Kamar N. Indications for and Barriers to Preemptive Kidney Transplantation: A Review. Transplant Proc (2010) 42(3):782–4. 10.1016/j.transproceed.2010.02.031 20430170

[35] Parmar A Vandriel SM Ng VL. Health-Related Quality of LIFE After Pediatric Liver Transplantation: A Systematic Review. Liver Transplant (2017) 23(3):361–74. 10.1002/lt.24696 28006876

[36] Kelly D Wray J. Non-Adherence and Transition Clinics. Best Pract Res Clin Gastroenterol (2020) 46-47:101687. 10.1016/j.bpg.2020.101687 33158474

[37] de Figueiredo CS Sandre PC Portugal LCL Mázala-de-Oliveira T da Silva Chagas L Raony Í COVID-19 Pandemic Impact on Children and Adolescents' Mental Health: Biological, Environmental, and Social Factors. Prog Neuropsychopharmacol Biol Psychiatry (2021) 106:110171. 10.1016/j.pnpbp.2020.110171 33186638 PMC7657035

[38] Digital N. Mental Health of Children and Young People in England, 2023 - WAVE 4 Follow Up to the 2017 Survey (2023).

