# Supplementary material for: Clinical Outcomes and Quality of Life of Patients Receiving Multi-Solid-Organ Transplants in Childhood Are Excellent: Results From a 20-Year Cohort Study
Source: Transpl Int. 2024 Aug 14;37:13372. doi: 10.3389/ti.2024.13372 (PMC11349566; doi:10.3389/ti.2024.13372)
Supplement: Supplementary file 1 [file DataSheet2.docx]

**Supplementary File 2: Results of the Thematic Analysis of Questionnaire Data on Quality of Life**

1. **Impact of Transplantation**
   1. Sense of self

I also see the person I was before my transplant (especially my heart transplant) as a completely different person to who I am now post-transplant. I struggle with that sometimes as I feel like that person died and sometimes I grieve for her - Patient 6, age 21

I would imagine if he [my son] had been older before his health problems began... he would also feel differently about how it relates to himself and who he is – Parent 4, age 15

Encourage children to be an ambassador of their health condition – Parent 1, age 8

- 1. Positive Impacts
     1. Overall improvement in quality of life

X’s quality of life has improved tremendously after her combined transplant. – Parent 10, age 9

Last 3 years since his transplants have been the best 3 years of his life, it felt like a bit of normality – Parent 24, age 16

This has been a life changing experience for our son – Parent 31, age 23

- - 1. Gratitude

We are so grateful the combined transplant was an option for her. - Parent 10, age 9

… the transplant was still so worth it … she wouldn't be alive without it – Parent 10, age 9

Transplant is actually good for you. I don't care about my kidney or anything, I'm just happy to be alive. – Patient 26, age 10

- - 1. Absence of fear / anxiety

Although he gets nervous when going to appointments etc, they are less frequent these days so he's not worried – Parent 26, age 10

Don't be scared. I like my doctor. – Patient 29, age 6

- - 1. Empowerment

Right now, I feel like I can take on the world and there is nothing stopping me. – Patient 8, age 18

- - 1. Improvement in physical symptoms

100% worth it, my child is now no longer vomiting 20+ times a day like before, he is able to walk now too – Parent 11, age 4

It [the transplant] was 17 years ago and he hasn't been in hospital since then. – Parent 31, age 23

- 1. Negative Impacts
     1. Physical Impacts

The biggest impact on me especially in the first 5 years post transplant was picking up childhood viruses and not being able to recover from them quickly. A normal cold could mean 3 weeks off school. – Patient 25, age 14

I get tired very quickly, even after only two car lengths- Patient 9, age 20

I get angry about all the hospital appointments, I hate that I have to go through all that. Annoyed at multiple pokes for blood tests each time. I hate going through all that - Patient 9, age 20

- - 1. Psychosocial Impacts

Coming to hospital makes me think of and remember the transplant and brings back those memories… Sometimes I just want to go outside and scream and cry. - Patient 9, age 20

I worry whether I will live long enough to fulfil my dreams to have children or whether I will have to have another transplant - and if it would work… often I question my own mortality - Patient 6, age 21

I used to find her crying alone because she was thinking of the transplant. – Parent 9, age 20

My son has a lot of emotions that he struggles to contain. He has never had control over what is done to him so he now tries to take control of as many situations as possible. He does not trust anyone, even us at times because we have had to do horrible procedures to him at home – Parent 29, age 6

The biggest problem he has had and still has is with his mental health. This has caused PTSD in our son and although he feels well physically, mentally he feels unstable and feels like he has missed out on his childhood. – Parent 31, age 23

- 1. Effects of the age at the time of transplant
     1. Benefits of transplanting at a young age

X received his transplants when he was very young, firstly aged 18 months and then 2 years… and thus it is all very normal day to day life for him… I think that for X, growing up whilst going through these experiences made them easier to bear as it was all so normal, and by the time he was old enough to be aware of what had happened to him, and of the ongoing treatments he had already done it all before many times. I would imagine if he had been older before his health problems began he would have had a vastly different experience, and would also feel differently about how it relates to himself and who he is. – Parent 4, age 15

Coming to hospital makes me think of and remember the transplant and brings back those memories. Particularly my second transplant as I was then old enough to remember it. – Patient 9, age 20

- - 1. Challenges of transplanting at a young age

We expect children to be resilient but the trauma of becoming so poorly and to need so much intervention at such a young age is underestimated. – Parent 31, age 23

1. **‘Normality’**
   1. Feeling different to others

I have always felt different to others my age but never really in a negative way - Patient 6, age 21

[I] never feel left out of things due to my transplant. – Patient 5, age 15

Sometimes I just want to go outside and scream and cry. I want to be like everyone else. – Patient 9, age 20

I question my own mortality which I am sure most people my age (21) don't – Patient 6, age 21

He [my son] struggles to socialise with his peers as he missed out on years of doing so due to long hospital admissions and hours on dialysis. He realises his condition makes him different to his peers and that he does not have the same gross motor skills as his friends – Parent 29, age 6

I often wonder if other children who are poorly from a very young have a similar experience to [my son] as they grow up. – Parent 4, age 15

They [transplanted children] notice changes in them and differences between them and other children. – Parent 1, age 8

- 1. ‘Normal Life’

Apart from a few aspects, I have a fairly normal life with some advantages - Patient 5, age 15

I worry whether I will live long enough to fulfil my dreams to have children – Patient 6, age 21

I'm very used to it all now – Patient 28, age 28

It is all very normal day to day life for him… I think that for [my son], growing up whilst going through these experiences made them easier to bear as it was all so normal – Parent 4, age 15

Last 3 years since his transplants have been the best 3 years of his life, it felt like a bit of normality – Parent 24, age 16

He lives a very normal life although there are certain jobs he can't do. – Parent 31, age 23

1. **Ongoing Care**
   1. Related to transplantation

I get angry about all the hospital appointments, I hate that I have to go through all that. Annoyed at multiple pokes for blood tests each time. - Patient 9, age 20

Teeth Health - transplant children struggle with better teeth health. Discolouration of teeth make them embarrassed. – Parent 1, age 8

- 1. Not directly related to transplantation

He has a peg so for those meds he won't take orally he gets them through this. – Parent 26, age 10

All medicine are done through the peg. – Parent 17, age 16

I have additional needs that affect my ability to independently understand and communicate about my transplant. I take liquid medication orally and rely on an adult to manage them. – Patient 25, age 14

My child has additional needs (autism, hearing and learning) which have a bigger impact on him than his transplant in terms of managing independently and understanding his health issues. He has liquid medication orally as a consequence which are drawn up and given by us as parents – Parent 25, age 14

- 1. Adulthood
     1. Healthcare services

Once my daughter reached adulthood and transitioned to adult care it became very difficult to coordinate appointments and to get attention from appropriate health care professionals for ongoing and new health issues because there is no one key person coordinating care like there was in paediatric care. – Parent 6, age 21

There has been little support for mental health except for a dedicated play therapist when he was a child. – Parent 31, age 23

- - 1. Other aspects of adulthood

Difficulty getting employment due to potential employers' concerns about impact of health issues on attendance or performance. – Parent 6, age 21

There are certain jobs he can't do – Parent 31, age 23

- - 1. Growing older

As they [transplanted children] grow, they notice changes in them and difference between them and other children. – Parent 1, age 8

I have found that as I grow older, I worry more and more about my future and the longevity of my transplanted organs – Patient 6, age 21

Especially as a person [a transplanted child] becomes older and is able to think more realistically about their life, it brings about some difficult questions. – Patient 6, age 21

- 1. Lack of support

Psychological support is very necessary… They need to be review by regular psychologist to stay confident and content. – Parent 1, age 8

No real support available for ongoing emotional or psychological problems such as anxiety or PTSD. – Parent 6, age 21

There has been little support for mental health except for a dedicated play therapist when he was a child. There is no support for him and we are having to pay private. The doctors are amazing but they cannot support him mentally. – Parent 31, age 23

1. **Suggestions for Improvement**
   1. Physical Health

Teeth Health - transplant children struggle with better teeth health. Discolouration of teeth make them embarrassed. Please set a set criteria for their teeth health, something for teeth whitening, or medicines that cause discolouration – Parent 1, age 8

- 1. Psychosocial Health
     1. Formal Support

Psychological support is very necessary, as they grow, they notice changes in them and difference between them and other children. They need to be review by regular psychologist to stay confident and content – Parent 1, age 8

- - 1. Peer Support

Encourage children to be an ambassador of their health condition and involve them in a group meeting where all transplant children meet, share their thoughts and comment about care they receive, doctors should be present as well so they can have a healthy discussion. – Parent 1, age 8
