# Supplementary material for: Clinical Outcomes and Quality of Life of Patients Receiving Multi-Solid-Organ Transplants in Childhood Are Excellent: Results From a 20-Year Cohort Study
Source: Transpl Int. 2024 Aug 14;37:13372. doi: 10.3389/ti.2024.13372 (PMC11349566; doi:10.3389/ti.2024.13372)
Supplement: Supplementary file 2 [file DataSheet1.docx]

**Supplementary File 1**

|  | |  |  |  |  |  |  |  |
| --- | --- | --- | --- | --- | --- | --- | --- | --- |
|  |  | **Total n=92** | **Liver and Kidney (n=72)** | | **Heart / Heart-Lung and Kidney (n=15)** | | **Pancreas and Kidney n=4** | **Multivisceral n=1** |
|  |  |  | **Combined n=53** | **Sequential n=19** | **Combined n=1** | **Sequential n=14** |  |  |
| Underlying Renal Disease (%) | **ARPKD** | 33 (36) | 31 (58) | 2 (10) | 0 (0) | 0 (0) | 0 (0) | 0 (0) |
|  | **PH1** | 15 (16) | 8 (15) | 7 (38) | 0 (0) | 0 (0) | 0 (0) | 0 (0) |
|  | **Metabolic Disease** | 5 (6) | 4 (8) | 1 (6) | 0 (0) | 0 (0) | 0 (0) | 0 (0) |
|  | **CAKUT** | 1 (1) | 1 (2) | 0 (0) | 0 (0) | 0 (0) | 0 (0) | 0 (0) |
|  | **Haemolytic Uraemic Syndrome** | 3 (3) | 1 (2) | 0 (0) | 0 (0) | 0 (0) | 2 (50) | 0 (0) |
|  | **Alagille Syndrome** | 2 (2) | 0 (0) | 2 (10) | 0 (0) | 0 (0) | 0 (0) | 0 (0) |
|  | **Ciclosporin Nephropathy** | 8 (9) | 0 (0) | 2 (10) | 0 (0) | 6 (43) | 0 (0) | 0 (0) |
|  | **Other Drug nephropathy** | 3 (3) | 0 (0) | 2 (10) | 0 (0) | 1 (7) | 0 (0) | 0 (0) |
|  | **Wilm’s Tumour** | 1 (1) | 0 (0) | 0 (0) | 1 (100) | 0 (0) | 0 (0) | 0 (0) |
|  | **Tubular Necrosis** | 2 (2) | 0 (0) | 0 (0) | 0 (0) | 2 (14) | 0 (0) | 0 (0) |
|  | **Tubular Interstitial Nephritis** | 1 (1) | 0 (0) | 0 (0) | 0 (0) | 1 (7) | 0 (0) | 0 (0) |
|  | **Glomerular Nephritis** | 1 (1) | 0 (0) | 0 (0) | 0 (0) | 1 (7) | 0 (0) | 0 (0) |
|  | **Other Renal Disease** | 17 (19) | 8 (15) | 3 (16) | 0 (0) | 3 (22) | 2 (50) | 1 (100) |
| Underlying Hepatic Disease (%) | **ARPKD** | 33 (45) | 31 (59) | 2 (10) | - | - | - | - |
|  | **PH1** | 15 (22) | 8 (15) | 7 (38) | - | - | - | - |
|  | **Metabolic Disease** | 5 (8) | 4 (7) | 1 (6) | - | - | - | - |
|  | **Hepatoblastoma** | 1 (1) | 1 (2) | 0 (0) | - | - | - | - |
|  | **aHUS** | 1 (1) | 1 (2) | 0 (0) | - | - | - | - |
|  | **Alagille Syndrome** | 2 (2) | 0 (0) | 2 (10) | - | - | - | - |
|  | **Biliary Atresia** | 2(2) | 0 (0) | 2 (10) | - | - | - | - |
|  | **Other Liver Disease** | 14 (19) | 8 (15) | 5 (26) | - | - | - | - |
| Underlying Cardiac Disease (%) | **Congenital Heart Disease** | 4 (27) | - | - | 0 (0) | 4 (33) | - | - |
|  | **Dilated Cardiomyopathy** | 5 (32) | - | - | 0 (0) | 5 (42) | - | - |
|  | **Restrictive Cardiomyopathy** | 1 (7) | - | - | 0 (0) | 1 (8) | - | - |
|  | **Doxorubicin Cardiomyopathy** | 1 (7) | - | - | 1 (100) | 0 (0) | - | - |
|  | **Other Cardiac Disease** | 4 (27) | - | - | 0 (0) | 4 (17) | - | - |
| Underlying Lung Disease (%) | **Fibrosing Lung Disease** | 1 (50) | - | - | - | 1 (50) | - | - |
|  | **Other** | 1 (50) | - | - | - | 1 (50) | - | - |
| Underlying Pancreatic Disease (%) | **Haemolytic Uraemic Syndrome** | 2 (50) | - | - | - | - | 2 (50) | - |
|  | **Other** | 2 (50) | - | - | - | - | 2 (50) | - |
| Underlying GI Disease (%) | **Primary Intrahepatic Cholestasis** | 1 (100) | - | - | - | - | - | 1 (100) |

Table S1: Table demonstrating the underlying pathologies for different transplanted organs. ARPKD = Autosomal Recessive Polycystic Kidney Disease, PH1 = Primary Hyperoxaluria Type 1, CAKUT = Congenital Abnormalities of the Kidney and Urinary Tract, aHUS = atypical haemolytic uraemic syndrome, GI = Gastrointestinal

|  | CLKT (n=53) | | SLKT (n=19) | | H/HLTKT (n=15) | | Pancreas and Kidney (n=4) | | Multi-visceral |
| --- | --- | --- | --- | --- | --- | --- | --- | --- | --- |
|  | **Liver** | **Kidney** | **Liver** | **Kidney** | **Heart/Heart-Lung** | **Kidney** | **Pancreas** | **Kidney** |  |
| Died with a functioning graft (%) | 2 (50) | 2 (17) | 2 (29) | 2 (40) | 2 (33) | 4 (44) | 0 (0) | 0 (0) | 1 (100) |
| Recurrent primary disease (%) | 0 (0) | 1 (8) | 0 (0) | 0 (0) | 0 (0) | 1 (12) | 0 (0) | 2 (100) | 0 (0) |
| Rejection while taking immunosuppression (%) | 0 (0) | 1 (8) | 0 (0) | 2 (40) | 2 (33) | 2 (22) | 1 (50) | 0 (0) | 0 (0) |
| Primary Non-Function (%) | 0 (0) | 1 (8) | 1 (13) | 0 (0) | 0 (0) | 0 (0) | 0 (0) | 0 (0) | 0 (0) |
| Vascular Occlusion (%) | 2 (50) | 0 (0) | 2 (29) | 0 (0) | 0 (0) | 0 (0) | 0 (0) | 0 (0) | 0 (0) |
| Other (%) | 0 (0) | 7 (59) | 2 (29) | 1 (20) | 2 (33) | 2 (22) | 1 (50) | 0 (0) | 0 (0) |
| Total | 4 | 12 | 7 | 5 | 6 | 9 | 2 | 2 | 1 |

*Table S2: Causes of Graft Loss in children Multi-solid-organ transplants. CLKT = Combined Liver and Kidney Transplant, SLKT = Sequential Liver and Kidney Transplant, H/HLKT = Heart/Heart-Lung and Kidney Transplant.*

|  | Total (n=92) | CLKT (n=53) | SLKT (n=19) | H/HLKT (n=15) | PKT (n=4) | Multivisceral (n=1) |
| --- | --- | --- | --- | --- | --- | --- |
| Multi-System Failure (%) | 5 (36) | 3 (75) | 1 (33) | 0 (0) | 0 (0) | 1 (100) |
| Respiratory Failure (%) | 2 (14) | 0 (0) | 1 (33) | 1 (17) | 0 (0) | 0 (0) |
| Hypertensive cardiac failure (%) | 1 (7) | 0 (0) | 0 (0) | 1 (17) | 0 (0) | 0 (0) |
| Other (%) | 6 (43) | 1 (25) | 1 (33) | 4 (66) | 0 (0) | 0 (0) |
| Total | 14 | 4 | 3 | 6 | 0 | 1 |

*Table S3: Table showing cause of death in children with CLKT and SLKT. CLKT = Combined Liver and Kidney Transplant, SLKT = Sequential Liver and Kidney Transplant, H/HLKT = Heart/Heart-Lung and Kidney Transplant, PKT = Pancreas and Kidney Transplant*

|  | **Patients** | **Parents** |
| --- | --- | --- |
| **About my Medicines 1** | 0.78 | 0.8 |
| **About my Medicines 2** | 0.81 | 0.76 |
| **My Transplant and Others** | 0.75 | 0.88 |
| **Pain and Hurt** | 0.71 | 0.85 |
| **Worry** | 0.83 | 0.95 |
| **Treatment Anxiety** | 0.82 | 0.95 |
| **How I look** | 0.45 | 0.83 |
| **Communication** | 0.87 | 0.95 |
| **Total** | 0.86 | 0.83 |

*Table S4: Cronbach’s coefficient alpha values for patient and parent questionnaire data.*
